# S10-4: Facilitating new interdisciplinary collaborations for active mobility: The Academic Observatory for Cycling and Active Mobilities (OUVEMA)

**DOI:** 10.1093/eurpub/ckae114.244

**Published:** 2024-09-26

**Authors:** Peter Gelius, Patrick Rérat, Bengt Kayser

**Affiliations:** Université de Lausanne, Switzerland; Université de Lausanne, Switzerland; Université de Lausanne, Switzerland

## Abstract

**Purpose:**

Although the current social, environmental, and health-related challenges call for a reflection of the place of mobility in tomorrow’s world, modern societies continue to be shaped by decades of motorization. Most European countries, including Switzerland, have not yet overcome this dependence. Exploring these issues requires finding ways to facilitate the collaboration between researchers from different backgrounds, e.g. geography, sport science, psychology, political science, and medicine, and a closer interaction between research and civil society.

**Project or policy description:**

Founded in 2020, the University Observatory for Biking and Active Mobility (OUVEMA) at the University of Lausanne has two missions: First, it aims to provide opportunities for collaboration of researchers from different disciplines to explore four main aspects of biking and active mobility: practices and behaviors; promotional policies; the built environment; and health issues. Specifically, the observatory provides an institutional framework for interdisciplinary research projects and publications related to active mobility, and connects researchers via its scientific and strategic committees. Second, the observatory seeks to connect research and civil society: It interacts with various civil society networks y in French-speaking Switzerland, as well as from European academic institutions pioneering research on the issue. To this end, OUVEMA organizes two annual series of lectures with changing topics, publishes a monthly newsletter sent to 2000 stakeholders, and disseminates information to media outlets.

**Conclusions:**

In the first three years of its existence, OUVEMA has shown that the topics of active mobility and biking open up the possibility for new collaborations between researchers in health, physical activity promotion, and other disciplines interested in human behavior and the built environment. OUVEMA might serve as a blueprint for the establishment of similar institutions across Europe. At the same time, it is crucial to further strengthen the collaboration between such centers of competence in active mobility across Europe to speed up the research and action required to tackle the upcoming global challenges.

